# Differences in drug resistance of HIV-1 genotypes in CSF and plasma and analysis of related factors

**DOI:** 10.1080/21505594.2023.2171632

**Published:** 2023-02-07

**Authors:** Jie Wang, Mei Li, Jungang Li, Renni Deng

**Affiliations:** Central lab, Chongqing Public Health Medical Center, Chongqing, China

**Keywords:** HIV-1, CSF, plasma, drug resistance, subtype

## Abstract

The emergence of HIV drug resistance seriously affects the quality of life of patients. However, there has been no extensive study of CSF resistance. The aim of this study is to evaluate common HIV-1 resistance in CSF and compare it with resistance in matched plasma, and analyse the influencing factors of cerebrospinal fluid drug resistance. The matched CSF and plasma samples of 62 HIV-1 patients were tested at one study site in China (Chongqing; 2019–2022). HIV genotyping and drug resistance was evaluated using the Stanford v8.7 algorithm. The diagnosis and treatment data and basic information were collected from the clinical case system, and the influencing factors of drug resistance mutations in CSF was obtained by variance analysis. CSF and matched plasma HIV-1 subtypes were confirmed in 62 patients, and the most frequent recombinant form was CRF07-BC (64.5%). Thirteen patients (21.0%) were detected with drug-resistant mutations, and the sites were consistent in both CSF and matched plasma. The drug-resistant ratios of untreated patients and treated patients were 5/51 (9.8%) and 8/11 (72.7%), respectively. The type with the highest mutation frequency was NNRTI, and no mutation was found in INSTI. Multivariate analysis indicated that ARV treatment was associated with CSF resistance (*P* < 0.001). The subtypes and drug resistance mutation sites are consistent in CSF and matched plasma samples of HIV-1 patients, and there is a correlation between ARV treatment and possible drug resistance, especially in CSF reservoirs. These findings highlight the concern about CSF drug resistance in HIV patients.

## Introduction

With the development of anti-HIV drugs and the widespread application of antiretroviral (ARV) therapy, the opportunistic infection rate and mortality rate of AIDS patients have been continuously reduced, and the quality of life has been significantly improved. However, the problem of ARV drug resistance has become increasingly prominent [1]. In high-income countries, the introduction of new effective and well-tolerated antiretroviral drugs has led to a decline in drug resistance. In Italy, the prevalence of any TDR mutation showed a consistent steep decline, decreasing from>14% in 2006 to about 7% in 2016 [2]. In contrast, resistance rates are increasing in low – and middle-income countries with limited resources, reaching 10.1% in East Africa and 11.0% in South Africa [3]. In China, resistance rates have also reached moderate prevalence levels (5–15%) in many areas, such as Guangxi (7.21%) and Shenyang (9.1%) [4,5]. The emergence of drug resistance seriously affects the long-term survival rate of HIV patients.

Meanwhile, studies have been shown that although ARV treatment can successfully inhibit HIV-1 in plasma, HIV-1 invading the central nervous system (CNS) can establish viral neural reservoirs through macrophages, microglia and lymphocytes [6,7]. The resultant emergence of relatively independent evolution of HIV-1, resulting in the cerebrospinal fluid (CSF) escape [8–10], has become a hidden danger to the failure of ARV treatment.

Since the replication and mutation of HIV-1 in the CNS are relatively independent, and the concentration of ARV drugs in different tissues varies, the drug concentration decreases after passing through the blood-brain barrier [11,12], as well irregular use of medication and other reasons lead to drug resistance. However, little is known about HIV-1 resistance in the CNS. Therefore, we compared HIV-1 drug resistance in CSF and in plasma, and studied the influencing factors of HIV-1 drug resistance in CSF.

## Patients and methods

### Patients

We retrospectively collected matched pairs of 68 HIV-1 patients who were admitted to Chongqing Public Health Medical Center from June 2020 to June 2022 and completed plasma CD4+ T cell counts, HIV RNA viral load, and CSF biochemical tests. CSF and plasma samples were tested for drug resistance of HIV genotypes. Among them, 6 patients had low CSF viral load and could not be tested for drug resistance. As a result, a total of 62 patients were included in this study, and all test reports were recorded. Demographic characteristics (sex, age) and clinical data (diagnosis time, CSF HIV RNA, total protein amount in CSF, CSF glucose, CSF white blood cell count, treatment regimen, plasma CD4 cell count) of these patients were collected and anonymously analysed. Thus, the usual requirement of written or oral informed consent is waived. This study has been approved by the Medical Ethics Committee of Chongqing Public Health Medical Center.

## HIV-1 gene amplification and drug resistance analysis

We amplified the nucleotide sequences of HIV-1 protease, reverse transcriptase and integrase using nested polymerase chain reaction (nest-pcr) method. A viral nucleic acid extraction kit (Jiangsu Shuoshi Company, China) was used to extract RNA from 00 μL plasma and CFS. The first round of PCR was performed using the HiScript® II One-Step RT-PCR Kit (Vazyme, Nanjing, China), followed by the second round of nested PCR using Ace Taq kit (Vazyme, Nanjing China). One-step RT-PCR and nested PCR was performed on a GeneAmp®9700PCR instrument (ABI, USA). The target bands were subjected to 1% agarose gel electrophoresis for validation, and the amplified product was sequenced. Sequences were then spliced and edited using the Seqman pro and Megalign7.0V software, and were submitted to the drug resistance database of Stanford University (https://hivdb.stanford.edu/hivdb/by-sequences/) for HIV-1 subtypes and resistance mutation analysis. PCR primers are listed in Supplementary Table 1.

## Statistical analysis

All data analyses were performed using the software SPSS 22.0. Quantitative variables are expressed as medians and IQR, and qualitative variables are expressed as percentages (%). The influencing factors (age, gender, duration of diagnosis, ARV treatment or not, plasma CD4 count, CSF HIV RNA, CSF HIV subtypes, CSF total protein, CSF glucose, CSF white blood cell count) related to drug resistance were selected for one-way analysis of variance. Variables with significant differences in one-way ANOVA were included in multi-factor ANOVA.

## Results

### Demographic characteristics

Overall, 62 HIV-1 patients with a median (IQR) age of 51 (38–64) years (51 ARV non- treated, including 49 naive; 11 ARV treated) were analysed (Table 1). CSF protein amount, glucose, and leukocyte content were comparable between patients who received or not received ARV treatment. The median CSF HIV-1 RNA (IQR) in ARV-non-treated and ARV-treated patients was 4.99 (4.47–5.72) and 4.47 (4.07–4.92) log10 copies/ml, respectively; the median plasma HIV-1 RNA (IQR) was 5.80 (5.48–6.32) and 5.30 (3.72–5.73) log10 copies/ml. The median CD4 cell count values (IQR) in non-treated and treated patients were 61 (35–106) and 76 (23–136) cells/mm^3^, respectively. All patients were infected with non-b subtype, and the most common recombinant form was CRF07-BC (64.5%). Nucleotide reverse transcriptase inhibitors (NRTIs) were the main drugs used in ARV-treated patients (Table 1).

**Table 1.** Characteristics of patients.

Characteristic	Total (*n* = 62)	ARV-non-treated patients (*n* = 51)	ARV-treated patients (*n* = 11)
Age, years, median (IQR)	51 (38–64)	56 (40–67)	45 (34–47)
Female, n (%)	12 (19.4)	10 (19.6)	2 (18.2)
Male, n (%)	50 (80.6)	41 (80.4)	9 (81.8)
Proteins of CSF mg/L, median (IQR)	474.67 (369.60–742.39)	479.82 (362.97–741.05)	469.52 (377.62–880.91)
Cerebrospinal fluid glucose mmol/L, median (IQR)	3.065 (2.40–3.59)	3.08 (2.33–3.63)	3.05 (2.75–3.54)
White blood cell count of CSF 10^6^/L, median (IQR)	6.5 (1–26.75)	6 (1–26.5)	8 (3–26.5)
CSF HIV-1 RNA, log10 copies/mL, median (IQR)	4.88 (4.23–5.65)	4.99 (4.47–5.72)	4.47 (4.07–4.92)
Plasma HIV-1 RNA, log10 copies/mL, median (IQR)	5.78 (5.30–6.25)	5.80 (5.48–6.32)	5.30 (3.72–5.73)
Plasma zenith HIV-1 RNA, log10 copies/mL, median (IQR)	5.83 (5.50–6.37)	5.84 (5.52–6.40)	5.78 (4.74–6.10)
Nadir CD4 count, cells/mm^3^, median (IQR)	52 (25–91)	55 (30–91)	21 (19–90.5)
CD4 count, cells/mm^3^, median (IQR)	63 (29.75–121.50)	61 (35–106)	76 (23–136)
CD8 count, cells/mm^3^, median (IQR)	364.5 (237–814.75)	365 (260–828.5)	283 (224.5–495)
CRF07-BC, n (%)	40 (64.5)^a^	35 (68.6)^a^	5 (45.5)
Current treatment, n (%)			
NRTIs+INSTIs+FIs	1 (1.61)	/	1 (9.09)
NRTIs+NNRTIs	3 (4.84)	/	3 (27.27)
NRTIs+INSTIs	3 (4.84)	/	3 (27.27)
NRTIs+PIs	2 (3.23)	/	2 (18.18)
FIs+INSTIs	1 (1.61)	/	1 (9.09)
NRTIs	1 (1.61)	/	1 (9.09)

Note: IQR, interquartile range; CSF, Cerebrospinal Fluid; NRTIs, nucleotide reverse transcriptase inhibitors, NNRTIs, nonnucleoside reverse transcriptase inhibitors; INSTIs, integrase strand transfer inhibitors; PIs, protease inhibitors; FIs, fusion inhibitors.

^a^Two patients of the ARV-non-treated group, as designated at the time of HIV genotyping, had received ARV treatment in the past.

## Drug resistance differences between cerebrospinal fluid and plasma genotypes

Next, the differences in drug resistance between CSF and plasma samples were analysed. Among the 13 patients with resistance mutation sites, the CSF and plasma subtypes were identical, mainly CRF07-BC (*n* = 5), and the mutation sites were consistent, and most of the patient had more than one mutation site. The mutation subtypes were mainly nonnucleoside reverse transcriptase inhibitor (NNRTI), nucleotide reverse transcriptase inhibitor (NRTI) and protease inhibitor (PI), and no mutation was found in integrase strand transfer inhibitor (INSTI). Among ARV-non-treated patients, 5/51 (9.8%) developed HIV-1 resistance mutations in both plasma and CSF: PI (*n* = 2), NNRTI (*n* = 3). The PI-related drug resistance mutation site was Q58E; the NNRTI-related mutation sites were mainly E138A/G and V179E (Table 2); Among patients receiving ARV treatment, 8/11 (72.7 %) had HIV-1 resistance mutations in both plasma and CSF: PI (*n* = 1), NRTI (*n* = 2), NNRTI (*n* = 1), NRTI+NNRTI (*n* = 3), NRTI+NNRTI+PI (*n* = 1). The PI-related drug resistance mutation sites are mainly Q58E, M46I, 54 V, V82A; the NRTI-related mutation sites are mainly 184 V/I, 65 R, Y115F, K70E/Q, 41 L, D67N, K219N; the NNRTI-related mutation sites were mainly E138A/G, V179E/D, K101E/Q, 106 M/I, Y181C, G190A, K103N, and 227 L (Table 2).

**Table 2.** Characteristics of HIV patients with drug resistance mutations.

Patient	Sex	Age (years)	Time since HIV diagnosis (weeks)	Total time under ARV treatment (weeks)	Time under current ARV treatment (weeks)	ARV	Subtype		HIV viral load (log_10_ copies/mL)		PI resistance mutations		NRTI resistance mutations		NNRTI resistance mutations		INSTI resistance mutations	
							CSF	Plasma	CSF	Plasma	CSF	Plasma	CSF	Plasma	CSF	Plasma	CSF	Plasma
1	F	36	0	0	0	naive	CRF07_BC	CRF07_BC	6.09	5.99	Q58E	Q58E	None	None	None	None	None	None
2	M	85	0	0	0	naive	CRF08_BC	CRF08_BC	5.62	5.48	None	None	None	None	E138A	E138A	None	None
3	M	69	26	0	0	naive	CRF08_BC	CRF08_BC	6.01	7.00	None	None	None	None	E138A	E138A	None	None
4	M	63	223	0	0	naive	CRF07_BC	CRF07_BC	4.53	6.03	Q58E	Q58E	None	None	None	None	None	None
5	M	27	120	52	0	no	CRF55_01B	CRF55_01B	4.76	5.53	None	None	None	None	E138G, V179E	E138G, V179E	None	None
6	F	44	26	8	8	3TC+TDF+EFV	CRF07_BC	CRF07_BC	4.16	5.69	None	None	None	None	V106M, V179D	V106M, V179D	None	None
7	M	26	120	120	120	3TC+TDF+EFV	CRF01_AE	CRF01_AE	5.04	5.60	None	None	K65R, Y115F, M184V	K65R, Y115F, M184V	K101E, Y181C, G190A	K101E, Y181C, G190A,	None	None
8	M	33	21	16	9	3TC+TDF+EFV	CRF07_BC	CRF07_BC	4.78	5.30	None	None	M41L, K65R, M184V	M41L, K65R, M184V	K101E, V106M, Y181C, G190A	K101E, V106M, Y181C, G190A	None	None
9	M	35	369	365	365	3TC+TDF+LPV/r	CRF01_AE	CRF01_AE	4.47	3.33	M46I, I54V, V82A	M46I, I54V, V82A	D67N, K70E, M184V	D67N, K70E, M184V	K103N, V106I, F227L	K103N, V106I, F227L	None	None
10	M	47	257	257	28	3TC+ABC+LPV/r	A	A	3.78	2.45	None	None	M184I, K219N	M184I, K219N	None	None	None	None
11	M	46	9	9	4	EVG/c/FTC/TAF+ABT	CRF01_AE	CRF01_AE	6.55	6.55	None	None	M184I	M184I	None	None	None	None
12	M	45	411	411	9	3TC+TDF	CRF01_AE	CRF01_AE	4.20	5.00	None	None	K70Q, Y115F, M184V	K70Q, Y115F, M184V	V106I	V106I	None	None
13	M	49	9	5	5	ABT+DTG	CRF07_BC	CRF07_BC	3.25	3.87	Q58E	Q58E	None	None	None	None	None	None

Note: 3TC, lamivudine; TDF, tenofovir disoproxil fumarate; EFV, efavirenz; LPV/r, lopinavir/ritonavir; ABC, abacavir; DTG, dolutegravir; ABT, albuvirtide; EVG/c/FTC/TAF, elvitegravir/cobicistat/emtricitabine/tenofovir alafenamide; F, female; M, male.

## Risk factors related to drug resistance in CSF

We assessed the association of CSF resistance with demographic and clinical factors (Table 3). Age, gender, duration of diagnosis, ARV treatment or not, plasma CD4 count, CSF HIV RNA, CSF HIV subtypes, CSF total protein, CSF glucose, CSF white blood cell count were grouped and analysed with one-way ANOVA. *P* values were 0.263, 0.689, 0.394, 0.000, 0.054, 0.480, 0.271,0.989, 0.807, 0.552, respectively. According to the 10% level of significance (*P* < 0.10), we found that ARV treatment/non-treatment and plasma CD4 counts were significant different in between groups. Therefore, we only performed multivariate analysis of variance with the above two factors, and the *P* values were 0.000 for ARV treatment and 0.370 for plasma CD4 count, suggesting receiving ARV treatment contributes significantly.

**Table 3.** Factors associated with HIV drug resistance in CSF.

Variables	Total (N)	n/N (%) with resistance	Univariate *P* value^a^	Multivariate *P* value^a^
Age (years)			0.263	
25–45	21	6/21 (28.57%)		
45–65	27	5/27 (18.52%)		
≥65	14	2/14 (14.29%)		
Sex			0.689	
male	50	11/50 (22.00%)		
female	12	2/12 (16.67%)		
Time since HIV diagnosis (months)			0.394	
0–6	42	5/42 (11.90%)		
6–24	3	2/3 (66.67%)		
24–60	6	3/6 (50.00%)		
≥60	11	3/11 (27.27%)		
Whether they are receiving ARV treatment			0.000***	0.000***
Yes	11	8/11 (72.73%)		
No	51	5/51 (9.80%)		
CD4 count (cells/mm3)			0.054*	0.370
<50	24	5/24 (20.83%)		
50–100	20	2/20 (10.00%)		
100–200	13	3/13 (23.08%)		
≥200	5	3/5 (60.00%)		
CSF HIV-1 RNA (log10 copies/mL)			0.480	
3–4	12	2/12 (16.67%)		
4–5	22	6/22 (27.27%)		
5–6	19	2/19 (10.53%)		
≥6	9	3/9 (33.33%)		
subtype			0.271	
A	1	1/1 (100.00%)		
C	2	0/2 (0.00%)		
CRF01-AE	11	4/11 (36.36%)		
CRF07-BC	40	5/40 (12.50%)		
CRF08-BC	7	2/7 (28.57%)		
CRF55-01B	1	1/1 (100.00%)		
Proteins of CSF (mg/L)			0.989	
150–500	34	7/34 (20.59%)		
500–1000	18	3/18 (16.67%)		
1000–2000	6	2/6 (33.33%)		
≥2000	4	1/4 (25.00%)		
Cerebrospinal fluid glucose (mmol/L)			0.807	
1–2	7	0/7 (00.00%)		
2–3	22	6/22 (27.27%)		
3–4	29	7/29 (24.14%)		
≥4	4	0/4 (00.00%)		
White blood cell count of CSF (10^6^/L)			0.552	
0–25	45	9/45 (20.00%)		
25–50	5	2/5 (40.00%)		
≥50	12	2/12 (16.67%)		

Note: *** and * represent the significance level of 1% and 10% respectively.

ARV, antiretroviral; CSF, Cerebrospinal fluid.

a One-way ANOVA was used to analyse the effect of different levels of a single variable on drug resistance, and multivariate ANOVA was used to analyse the two variables with significant differences. P-values with significant differences are shown in bold.

## Discussion

This study compared the PI, NRTI, NNRTI, and INSTI resistance profiles in CSF and plasma of HIV-1 patients for the first time, and demonstrated that CSF and plasma have consistent drug resistance mutations. In addition, the statistical analysis of the factors associated with drug resistance in CSF confirmed that whether or not to receive ARV treatment was significantly associated with the resistance.

Due to the lack of correction function of HIV reverse transcriptase at replication, physiological fluctuations of dNTP pools and asymmetric error repair, the mutation rate of replication reaches 10^−4^ to 10^−5^, which makes it almost impossible to determine the fidelity of a single round of replication [13]. Although ARV therapy can effectively attenuate HIV-1 replication in the peripheral system, HIV-1 virus can still maintain chronic and persistent replication in the CNS due to the protective effect of the blood-brain barrier [14,15]. This group of HIV-1 continuously replicates and evolves relatively independently in the CNS, and can be transmitted to the peripheral blood through microglia and T lymphocytes [16,17], thereby causing peripheral viral rebound and drug resistance. All drug-resistant patients in this study could be detected with a large amount of HIV-1 in CSF and plasma, which showed the same subtype profiles. The consistent drug resistance profile also suggested that the HIV-1 in CSF and peripheral blood may be of the same origin. This was also confirmed by the phylogenetic analysis (Supplementary Figure 1).

Currently, the international recommended ARV treatment regimen is combination of NRTIs and third-class drugs, and the third-class drugs can be NNRTIs or enhanced PIs or INSTIs; or compound single-tablet regimens (STR) [18–20]. Long-term fixed drug treatment regimens are also prone to lead to common drug resistance mutations. For example, in our study, The NRTIs 3TC and TDF in multiple ARV treatment regimens are prone to cause 184 V/I and 65 R mutations; NNRTI EFV is prone to generate Y181C, G190A and K101E mutations; PI LPV/r appears to lead to M46I and V82A mutations [21,22], and we also detected additional PI-related mutation Q58E (3/13), which is consistent with the drug resistance profile in China [5,23]. Maybe there is an intrinsic evolutionary rate within the host that contributes to HIV drug resistance, but widespread use of low genetic barrier antiretrovirals also contributes to the development of drug resistance [21]. The incidence of drug resistance in middle and low-income countries is rising due to the antiretroviral treatment implementation with low genetic barrier regimens based on 2NRTIs+NNRTI. For example, the 3TC of NRTIs, which has the lowest genetic barrier to drug resistance, may take only two weeks to develop drug-resistant mutations [20]. While INSTIs, as one of the latest ARV drugs used in developed countries, have higher genetic barriers to resistance compared with NRTIs and NNRTIs. Since it is still in the initial stage of application of INSTIs in China, no related drug resistance mutations have been found yet in this study [24].

Emerging evidence indicate that certain antiretroviral drugs have low penetration in the CNS [25,26], and that insufficient drug penetration and/or lack of compliance predisposes to the emergence of CSF resistance [27,28]. However, the current research on HIV-1 in CSF is mostly about the effect of HIV RNA on neurocognitive dysfunction, and little is known on the influencing factors of HIV-1 drug resistance mutation in CSF. Therefore, our study assessed the correlation between resistance and each variable and demonstrated that CSF resistance may be only related to whether or not to receive ARV treatment. There was no correlation between resistance and factors including the duration of HIV diagnosis, HIV RNA in CSF, subtype, CSF total protein, CSF glucose, CSF white blood cell count, or CD4 count in plasma. Due to the limited amounts of specimens and detection methods, our results failed to detect a deeper underlying correlation between CSF resistance and influencing factors, or other relevant influencing factors. Further efforts need to be put forward in future explorations.

In conclusion, the present study demonstrates consistent PI, NRTI, NNRTI and INSTI resistance profiles in the CNS and matched plasma of HIV-1 patients. Meanwhile, the evaluation on the influencing factors of CSF drug resistance shows that receiving ARV treatment may increase the incidence of HIV-1 drug resistance in CSF.

## Supplementary Material

Supplemental Material

## Data Availability

The data sets analysed during the current study are available from the corresponding author on reasonable request.
