# Supplementary material for: Differences in drug resistance of HIV-1 genotypes in CSF and plasma and analysis of related factors
Source: Virulence. 2023 Feb 7;14(1):2171632. doi: 10.1080/21505594.2023.2171632 (PMC9908293; doi:10.1080/21505594.2023.2171632)
Supplement: Supplemental Material [file KVIR_A_2171632_SM6259.zip › supplementary/Supplementary Figure 1.docx]

Phylogenetic tree of PR and RT regions

P represents plasma and C represents cerebrospinal fluid. Phylogenetic analysis was used to evaluate the relationships between CFS and matched plasma. There was no difference in HIV pol sequences from CSF and matched plasma.

Phylogenetic tree of IN region

P represents plasma and C represents cerebrospinal fluid. Phylogenetic analysis was used to evaluate the relationships between CFS and matched plasma. There was no difference in HIV pol sequences from CSF and matched plasma.
