# Supplementary material for: Differences in drug resistance of HIV-1 genotypes in CSF and plasma and analysis of related factors
Source: Virulence. 2023 Feb 7;14(1):2171632. doi: 10.1080/21505594.2023.2171632 (PMC9908293; doi:10.1080/21505594.2023.2171632)
Supplement: Supplemental Material [file KVIR_A_2171632_SM6259.zip › supplementary/Supplementary Table 1.docx]

Supplementary Table 1.

Primers for amplification and sequence analysis of HIV-1 Protease（PR）and Reverse transcriptase（RT）region

| Procedure | Primers | Position^a^ | Length of target fragment | Sequences (5'-3') |
| --- | --- | --- | --- | --- |
| First round | 2029F | 2029–2050 | 1501 | TGGAAATGTGGRAAGGAAGGAC |
|  | 3529R | 3529–3505 |  | GCTAyyAAGTCTTTTGATGGGTCAT |
| Second round | 2249F | 2249–2266 | 1273 | CTTCCCTCARATCACTCT |
|  | 3521R | 3521–3504 |  | GTCTTTTGATGGGTCATA |

Primers for amplification and sequence analysis of HIV-1 Integrase（IN） region

| Procedure | Primers | Position^a^ | Length of target fragment | Sequences (5'-3') |
| --- | --- | --- | --- | --- |
| First round | 4007F | 4007–4030 | 1213 | GCAGGATTCRGGATYAGAAGTAAA |
|  | 5219R | 5219–5243 |  | CCTAGTGGGATGTGTACTTCTGAAC |
| Second round | 4063F | 4063–4080 | 1157 | TCATTCARGCACAACCAG |
|  | 5219R | 5219–5243 |  | CCTAGTGGGATGTGTACTTCTGAAC |

^a^ Nucleotide positions with reference to HIV HXB2 strain (GenBank accession number: K03455)
