# Supplementary material for: Differences in drug resistance of HIV-1 genotypes in CSF and plasma and analysis of related factors
Source: Virulence. 2023 Feb 7;14(1):2171632. doi: 10.1080/21505594.2023.2171632 (PMC9908293; doi:10.1080/21505594.2023.2171632)
Supplement: Supplemental Material [file KVIR_A_2171632_SM6259.zip › supplementary/Supplementary Table 2.docx]

Supplementary Table 2.

Factors associated with HIV drug resistance in plasma

| Variables | Total (N) | n/N (%) with resistance | Univariate P value^a^ | Multivariate P value^a^ |
| --- | --- | --- | --- | --- |
| Age(years) |  |  | 0.263 |  |
| 25-45 | 21 | 6/21(28.57%) |  |  |
| 45-65 | 27 | 5/27(18.52%) |  |  |
| ≥65 | 14 | 2/14(14.29%) |  |  |
| Sex |  |  | 0.689 |  |
| male | 50 | 11/50(22.00%) |  |  |
| female | 12 | 2/12(16.67%) |  |  |
| Time since HIV diagnosis(months) |  |  | 0.394 |  |
| 0-6 | 42 | 5/42(11.90%) |  |  |
| 6-24 | 3 | 2/3(66.67%) |  |  |
| 24-60 | 6 | 3/6(50.00%) |  |  |
| ≥60 | 11 | 3/11(27.27%) |  |  |
| Whether they are receiving ARV treatment |  |  | **0.000*** | **0.000*** |
| Yes | 11 | 8/11(72.73%) |  |  |
| No | 51 | 5/51(9.80%) |  |  |
| CD4 count, (cells/mm^3^) |  |  | **0.054*** | 0.370 |
| <50 | 24 | 5/24(20.83%) |  |  |
| 50-100 | 20 | 2/20(10.00%) |  |  |
| 100-200 | 13 | 3/13(23.08%) |  |  |
| ≥200 | 5 | 3/5(60.00%) |  |  |
| Plasma HIV-1 RNA, (log10 copies/mL) |  |  | 0.862 |  |
| 3-5 | 12 | 4/12(33.33%) |  |  |
| 5-6 | 28 | 6/28(21.43%) |  |  |
| 6-7 | 21 | 2/21(9.52%) |  |  |
| ≥7 | 1 | 1/1(100.00%) |  |  |
| subtype |  |  | 0.271 |  |
| A | 1 | 1/1(100.00%) |  |  |
| C | 2 | 0/2(0.00%) |  |  |
| CRF01-AE | 11 | 4/11(36.36%) |  |  |
| CRF07-BC | 40 | 5/40(12.50%) |  |  |
| CRF08-BC | 7 | 2/7(28.57%) |  |  |
| CRF55-01B | 1 | 1/1(100.00%) |  |  |
| Total plasma protein (mg/L) |  |  | 0.295 |  |
| 40000-60000  60000-70000  70000-80000  ≥80000 | 10  19  26  7 | 1/10(10/00%)  3/19(15.79%)  7/26(26.92%)  2/7(28.57%) |  |  |
| Plasma glucose (mmol/L) |  |  | 0.686 |  |
| 3-4 | 6 | 2/6(33.33%) |  |  |
| 4-5 | 25 | 5/25(20.00%) |  |  |
| 5-6 | 23 | 3/23(13.04%) |  |  |
| ≥6 | 8 | 3/8(37.50%) |  |  |
| White blood cell count of Plasma (10^6^/L) |  |  | 0.140 |  |
| 0-1000 | 3 | 0/3(0.00%) |  |  |
| 1000-2000 | 14 | 3/14(21.43%) |  |  |
| 2000-3000 | 17 | 3/17(17.65%) |  |  |
| 3000-4000 | 19 | 3/19(15.79%) |  |  |
| 4000-5000 | 4 | 1/4(25.00%) |  |  |
| ≥5000 | 5 | 3/5(60.00%) |  |  |

*** and * represent the significance level of 1% and 10% respectively

ARV, antiretroviral; CSF, Cerebrospinal fluid

^a^ One-way ANOVA was used to analyze the effect of different levels of a single variable on drug resistance, and multivariate ANOVA was used to analyze the two variables with significant differences. P-values with significant differences are shown in bold.
